# bTB eradication in Ireland: where to from here?

**DOI:** 10.1186/s13620-023-00239-8

**Published:** 2023-07-04

**Authors:** Simon J. More

**Affiliations:** Centre for Veterinary Epidemiology and Risk Analysis, UCD School of Veterinary Medicine, University College Dublin, Belfield, , Dublin, D04 W6F6 Ireland

**Keywords:** Bovine tuberculosis, Eradication, Ireland, Uncertainty, Wildlife, Risk-based approaches, Industry commitment, Programme governance

## Abstract

**Background:**

In an earlier paper from 2019, this author concluded that successful eradication of bovine tuberculosis (bTB) from Ireland by 2030 would be unlikely, given control strategies in place at that time plus the addition of badger vaccination. He argued that additional measures will be needed, broadly focusing on bTB risks from wildlife, risk-based cattle controls, and industry commitment. This paper considers these points in further detail.

**Main text:**

Ongoing monitoring of the badger vaccination programme (which is progressively being rolled out nationally) and associated research will be critical, with a focus both on programme inputs and outcomes. The direct contribution of cattle movements to bTB restrictions in Ireland has been evaluated. However, it is the indirect role of cattle movements in bTB restrictions that is likely of greater importance, particularly towards the latter phase of the eradication programme. In other national programmes, a range of risk-based approaches have been used to address the challenge of residual infection in cattle (that is, the presence of animals with persistent but undetected infection), and similar approaches are needed in Ireland. A number of authors have highlighted the critical importance of industry commitment to programme success, and the key role of programme governance to achieving this. In this commentary, the author briefly considers experiences from Australia and New Zealand in this regard. The author also reflects on the challenge of uncertainty in decision-making, the relevance to Ireland of lessons from other countries, and the potential contribution of new methodologies in support of the national programme.

**Conclusions:**

‘The tragedy of the horizon’ was a term first used in the context of climate change, referring to the costs imposed on future generations that the current generation has no direct incentives to fix. This concept is equally relevant to bTB eradication in Ireland, where current decisions will have long-term consequences for future generations, including both the general public (through the Exchequer) and future Irish farmers.

In recent years, there has been a deterioration to the national bovine tuberculosis (bTB) situation in Ireland. From an historic low herd incidence rate of 3.27% in 2016, this metric has risen to 4.33% in 2021, and 4.31% in 2022. Concurrently, total expenditure of the programme has increased from €82 million in 2015 to €97 million in 2020, with the Exchequer being the largest contributor (rising from 47.5% to 58.6% of overall programme costs over this period) [1]. To give a clearer sense of the magnitude of these costs, cumulative programme costs over the last 20 or so years now approximate €2 billion which, if condensed into a single year, would represent approximately 0.4% of annual GDP in 2023.

In an earlier review [2], I concluded that current strategies plus badger vaccination would not be sufficient to successfully eradicate bTB from Ireland by 2030. Further, I noted that adequate information was available, both from research and international experience, to suggest that additional measures were needed, broadly focusing on bTB risks from wildlife, risk-based cattle controls, and industry commitment. The need for a multi-pronged approach was also endorsed by national programme managers at the international *M. bovis* conference that was held in Galway, Ireland, in June 2022. This commentary briefly considers these points in further detail.

Wildlife play a role in the epidemiology of bTB in cattle in many countries, and represent a key challenge to eradication efforts. In Ireland, there is robust evidence in support of the efficacy of field badger vaccination by injection, and – with uncertainty – of the positive contribution of badger vaccination to national eradication efforts. For some years now, there has been a national roll-out of a badger vaccination programme, seeking to progressively replace badger culling. Ongoing monitoring and research will be critical, both of programme inputs (particularly vaccine coverage, noting the enormous challenges associated with the national vaccine roll-out, both to implement and maintain) and outcomes (including the ecology of, and the dynamics of infection in, vaccinated badger populations), and to determine reasons for success and failure. In most areas of Ireland, there has been no evidence to date to suggest that deer (generally Sika, *Cervis nippon*, an introduced species, and Sika hybrids) are a bTB maintenance host. In Co. Wicklow, however, and potentially elsewhere, this may change (or is perhaps already changing), due to the influence of those factors that facilitate the establishment and perpetuation of bTB in deer. Ongoing ecological and epidemiological research in deer will be important.

With respect to cattle, residual infection (that is, the presence of animals with persistent but undetected infection) is a recognised feature of bTB epidemiology, and a further challenge to eradication. This phenomenon occurs within test-based eradication programmes throughout the world, and is the key rationale for ongoing efforts towards improved diagnostic tests. Because there is very substantial cattle movement in Ireland, there is no doubt that ongoing commerce will result in the movement of some cattle with residual infection. This has been evaluated in a range of studies, including an estimate of 7.4% of bTB restrictions that are directly attributable to the recent introduction of an infected animal [3]. However, it is the indirect role of cattle movement in bTB restrictions that is likely of greater importance, particularly towards the latter phase of the eradication programme, as bTB levels falls and infection-free areas become increasing important. Here, cattle movement has the potential to ‘seed’ infection to areas previously non-infected, leading to the establishment of infection in local cattle and wildlife. Other national programmes have used a series of risk-based approaches to address the challenge of residual infection, including the differentiation of management units (regions, herds) on the basis of bTB risk, and the implementation of control decisions, including movement controls, with the aim to prevent bTB transmission from management units of higher to lower risk. Similar approaches are needed in Ireland, noting that substantial knowledge of bTB risk, based on local research, is available.

A number of authors have highlighted the critical importance of industry commitment to programme success, and the key role of programme governance to achieving this. In Australia, fundamental change to programme governance were introduced in 1984 (some 14 years after the start of their national programme) coinciding with rising industry opposition to the use of mass destocking to tackle bTB (and brucellosis) in difficult northern areas. With these changes, industry subsequently played a central role in programme decision-making at all levels. The trust built between government and industry during this and associated programmes, and the lessons learned, has played a key role in the establishment and ongoing operation of animal health programmes in Australia, including Animal Health Australia. In New Zealand, TBfree has been the statutory management agency responsible for bTB eradication since 2013. Working in partnership with government and the livestock industries, TBfree operates within a defined cost envelope that encompasses all programme costs, including compensation. A legally agreed costing model is in place, underpinned by national legislation, based on formal recognition of beneficiaries (those who benefit from bTB control) and exacerbators (those who, through their action or inaction, exacerbate the problem). Strategic planning, robust science and programme success are each recognised as the key means to mitigate the financial liabilities borne by industry (and to a lesser extent government). This partnership model, in place in New Zealand since 1993, has overseen substantial and sustained progress towards bTB eradication. Although there is now greater stakeholder involvement in Ireland following the establishment of the national TB Forum in 2018, programme governance remains firmly under the remit of government. Further, current cost- and responsibility-sharing arrangements in Ireland do not accurately reflect the balance of private goods (78% share, primarily market access) and public goods (22% share, primarily increased tax returns arising from increased market access) that derive from bTB eradication, as determined in a recent national review [4].

I highlight three key points relevant to the issues raised previously:

- Firstly, decision-makers need to take care when dealing with uncertainty (that is, imperfect or unknown knowledge) in the context of bTB eradication. Decisions will always be made in the context of some degree of uncertainty, and it is for this reason that uncertainty *per se* is rarely justification for inaction. Here, we need to distinguish uncertainties that have the potential to lead to a fundamental – compared to an incremental – shift in knowledge about bTB epidemiology and eradication options. To illustrate, there is currently considerable uncertainty about the relative contribution of different infection sources (cattle movement, residual infection, badgers, deer etc.), and an improved national understanding will certainly be helpful (but is currently technically challenging, given available methods). However, this knowledge, once available, is unlikely to challenge the principle – accepted internationally – that all sustaining infection sources must concurrently be addressed for national bTB eradication efforts to be effective.
- Secondly, there has been a view that lessons from other countries cannot readily be extrapolated to Ireland, given differences in farming systems, climate, wildlife hosts etc. Certainly there are differences; however, it is also clear that the broad principles of bTB eradication have been similar across all environments where bTB has proved (or is proving) successful. This is particularly the case with respect to cattle controls and industry commitment. For example, no country has yet been able to eradicate bTB without introducing comprehensive, risk-based controls on cattle movement.
- Thirdly, a range of promising new methodologies are now emerging, including whole genome sequencing, mathematical modelling and machine learning. These are now being implemented in Ireland, and have the potential to provide greater clarity in current areas of scientific uncertainty.

In the current agri-political environment in Ireland, it can be very challenging to make difficult or unpopular decisions. Given existing costing arrangements and the current national bTB trajectory, in part this is because decisions made now may have only a limited positive impact on the current generation of Irish farmers, certainly over the shorter term. We are constrained therefore to those decisions that are politically possible. However, with reference to bTB eradication, current decisions will have long-term consequences for future generations, including both the general public (through the Exchequer) and future Irish farmers. This is because these decisions will influence both ‘time to eradication’ and overall programme costs (which have the potential to become very substantial indeed). This concept, called ‘the tragedy of the horizon’ was first used by the Governor of the Bank of England in the context of climate change [5], and refers to the costs imposed on future generations that the current generation has no direct incentives to fix. The tragedy of the horizon is equally relevant to bTB eradication in Ireland.

## Data Availability

Not applicable.
